# The secret garden? Elite metropolitan geographies in the contemporary UK

**DOI:** 10.1111/1467-954X.12285

**Published:** 2015-06-12

**Authors:** Niall Cunningham, Mike Savage

**Affiliations:** 1 Durham University; 2 LSE

**Keywords:** social class, Great British Class Survey, elites, spatial analysis, Geographical Information Systems (GIS), spatial inequality

## Abstract

There is an enduring, indeed increasing awareness of the role of spatial location in defining and reinforcing inequality in this country and beyond. In the UK, much of the debate around these issues has focussed on the established trope of a long-standing ‘north-south divide’, a divide which appears to have deepened in recent decades with the inexorable de-industrialisation of northern Britain presented in stark counterpoint to the burgeoning concentration of wealth in London and the south-east, driven by the financial and ancillary services sectors. Due to a lack of available data, such debates have tended to focus solely on economic inequalities between places, and until now there was little understanding of how these disparities played out in the social and cultural domains. This paper significantly advances our understanding of the true meaning of spatial inequality in the UK by broadening that definition to encompass not only the economic, but also the social and cultural arenas, using data available from the BBC's Great British Class Survey experiment. We argue that these data shine a light not only on the economic inequalities between different parts of the country which existing debates have already uncovered but to understand how these are both reinforced and mediated across the social and cultural dimensions. Fundamentally, we concur with a great many others in seeing London and the south-east as a vortex for economic accumulation but it is also much more than that; it is a space where the coming together of intense economic, social and cultural resources enables the crystallisation of particular and nuanced forms of elite social class formations, formations in which place is not incidental but integral to their very existence.

## Introduction

Inequality has always been a central concern of sociology and while sociologists have stressed the importance of an awareness of the spatial context within which social life occurs, academics from within the discipline have historically tended to analyse the issue from a largely aspatial perspective. Furthermore, while there is consensus on growing inequality in the United Kingdom in general terms (Dorling and Rees, 2003; Wilkinson and Pickett, 2009; Dorling, 2011) as well as an understanding of the powerful spatial patterns of persistence in the distribution and reproduction of disadvantage over the long term (Dorling *et al*., 2000; Gregory, 2009), the corresponding geography of affluence as a field of intellectual study has been left relatively fallow until very recently (Burrows, 2013a, 2013b). To some extent this has been reflective of the traditional concerns of sociology and a lack of sufficiently-detailed data (Lobao *et al*., 2007), but it was also a product of an innate cautiousness within the discipline over the adoption of technologies such as Geographical Information Systems (GIS) which were previously perceived as ‘socially-constructive’ (Harvey and Chrisman, 1998). However, Savage and Burrows (2007) have more recently argued in favour of the need to embrace new methodological approaches within sociology in order to extend the discipline's reach and relevance.

The Great British Class Survey (GBCS) provides a powerful resource for the spatial analysis of the elite in British society, as due to its unprecedented scope it enables us not merely to identify a group hitherto absent from existing influential social schema due to the limitations of previous samples (Penn, 1981; Savage and Williams, 2008), but to anatomize in remarkable detail the ‘micro-classes’ (Butler and Savage, 1995; Devine, 2010; Grusky and Weeden, 2001, 2008) that exist within this social formation. Within the elite there exists a number of smaller groupings, striking for the ways in which their spatial locations can be seen to be emblematic of a wider sense of ‘habitus’ in which the connection between objective social structures and individual responses or choices is mediated (Bourdieu, 1984). In this way we can provide empirical substantiation to the definition of an ‘elite’ as an inherently recursive concept (Dorling, 2013).

## Elites and space: an intellectual framing

It has become a *sine qua non* for current political debates on social change to acknowledge that inequalities between the richest and poorest are increasing, and that such inequalities have profound social consequences (eg Dorling, 2011; Wilkinson and Pickett, 2009; Wacquant, 2008; Atkinson *et al*., 2013; Hills *et al*., 2013). While such texts reflect a vital and necessary focus, it is also apparent that within the social sciences there has been until very recently, a relative neglect of those who occupy the upper echelons of the socio-economic hierarchy, and very specifically on their geographies (Burrows, 2013a). In addressing these issues it is clear that a ‘social exclusion’ framework which focuses solely on those at the bottom of the socio-economic spectrum and defines the issues in terms of the factors which cause disadvantage and which operate to exclude them from the social body, is deficient. This can act to construct a narrative solely around the characteristics and problems of the most disadvantaged people and places, and does not address how the power and privileges of the advantaged are organized.

To date, little academic attention has sought to counter-balance this by developing our understanding of the issues around the geographies of privilege and affluence. This lacuna has been the product not just of political framing but is also linked to a lack of available data with comprehensive and sophisticated questions on the socio-cultural practices and preferences of this group (though see Hay and Muller, 2012). As things stand we have a very limited empirical understanding of the geographies of privilege across the country as a whole and despite the influential interventions of radical geographers such as Dorling who has mapped a considerable range of indicators of privilege and power, these have not, to date, allowed a mapping of the kinds of measures of cultural and social capital that have been elaborated in recent sociological research and offered a powerful intervention in the field of ‘cultural class analysis’ (eg Bennett *et al*., 2009). This paper contributes to a rebalancing of these debates in presenting the most detailed sociological mapping of elite practices in the UK currently available.

A starting point is that we need to more fully understand the remaking of elite advantaged groups in relation to the seismic economic restructuring of this country which occurred in the last decades of the twentieth century and which juxtaposes deindustrialization of northern Britain to the burgeoning wealth of London and the south-east, where the financial services sector has become an ever-increasing component of the regional and by extension, national economy (see, in general terms, Bowman *et al*., 2012; Dorling, 2011; Ertürt *et al.*, 2011). The geographical aspects of this shift are familiar, being organized around a ‘north-south’ narrative, with much intellectual energy focused on measuring deprivation as a means of charting the problematic features of these processes. The intensity of media coverage around these issues (BBC, 2014) would suggest that such dynamics are a recent phenomena, but the roots of such reductive binaries can be traced back much further (Campbell, 2004). If we take the long view, the UK has always been dominated by a London commercial and mercantile elite, whose economic interests were forever outward towards the empire rather than north towards the industrial heartland (Rubinstein, 1993). In this paper we seek to elaborate this argument about London's role as an elite vortex.

However, we insist at the outset that our argument in no way suggests that London is a land of milk and honey for all its residents and the inequalities evident across the country are at least as pronounced at intra-city level within London. For Sassen (1991), increasing social polarization in London is the direct outcome of ‘hourglass’ (Nolan, 2001) macroeconomic dynamics which have seen the burgeoning of highly paid work in the financial services sector matched by a ballooning of the workforce at the opposite end of the employment spectrum in the service sector, while opportunities for employment and advancement in the middle ranges for skilled workers have evaporated due to the collapse of the UK's manufacturing base in the second-half of the twentieth century. This has led to a growing army of people in poorly rewarded and insecure work, a new social division of labour which as Butler and Watt (2007) have found, has profound spatial implications as this group is both essential to, yet increasingly marginalized by the ongoing capitalist project in London (Massey, 2005). For Hamnett (2003), a more appropriate metaphor is perhaps the cocktail glass rather than the hourglass, for he sees the problem not as one of a glut of low-quality work options, but of any meaningful options for a rump of long-term economically inactive people who effectively lie beyond the impact of such labour market impulses. Massey (2007) makes an important intervention by bringing the inter-regional and intra-regional debates together by exposing the north–south dichotomy as what she sees as almost a form of spatial false consciousness which serves to obscure poverty and inequality within the very ha-has of the nation's neoliberal elite in London and the south-east through an obsessive focus on divisive north–south competition. In our view, revealing the distinctive elite character of a minority of London's population allows these arguments to be elaborated further.

It might be suggested that another factor acting to shroud inequality in London and the south-east is the particular transnational character of wealth in the city: this is a central feature of the global city around which differing authors agree. Sassen (1991) has seen this as the outcome of processes of economic dispersal which, ironically, have led to the development of major financial centres to service the requirements of a globalized economy in which the relationship between big corporations and national governments is one of inexorable, increasing subservience (Butler and Watt, 2007). This line of argument points to the emergence of a placeless, global elite, facilitated by a new capitalist order in which information is now the most powerful currency, the exchange of which has been facilitated by rapid technological innovation (particularly the Internet), and necessitated by exactly the sort of decentralizing forces of production identified by Sassen (1991). Manual Castells memorably articulates this vision in *The Rise of the Network Society* (1996), but in the case he presents one hears powerful echoes of that made by Melvin Webber (1964) more than three decades earlier in a paper in which he argued that ‘intellectual elites’ constituted

spatially dispersed, *nonplace* communities. These folk approximate the true cosmopolities for whom territorial distance is a minor barrier to interaction and whose professional social communities are the least shaped by territorialism. For these, social propinquity is least dependent upon spatial propinquity.

However, our argument is actually that London should not simply be seen as the centre of a placeless elite, and that in fact we can detect distinctive forms of elite territoriality at work here (and see further Burrows and his colleagues, 2013b, on London's ‘Super-Rich’).

The source for our work is the Great British Class Survey (GBCS). We acknowledge at the outset that the methodological issues in deploying this are intense. Savage *et al*. (2013) show that GBCS data is biased towards a younger sample who are more likely to have attended university than is representative of the population for the UK at large. For our purposes here, however, this offers a striking opportunity as it provides a powerful lens to explore in spatial and statistical terms a group which has been neglected in sociological studies in recent decades, notably the small GBCS ‘Elite’ which our earlier work has shown was differentiated from the six other classes in terms of their striking possession of economic capital (and to a lesser extent, their considerable holdings of cultural and social capital).

The fact that we have access to such detailed information on this grouping confounds sociological and public conceptions of the elite as a sector of British society who tend to reveal little about themselves, their habits and their resources. Rather, the dataset is testament to Massey's (2007: 120) assessment that the literal and metaphorical location of this group in British society is in fact ‘a space of introspection and absorption’. The unprecedented level of enthusiasm which this cadre has itself shown for the GBCS suggests that we may have to fundamentally reappraise the social and cultural motifs of modesty and reserve which have traditionally been applied to upper classes sociology (see in general, on the ‘modesty’ of the successful, Miles *et al*., 2011).

This paper proceeds by initially seeking to explicate the sample skew in the GBCS survey through linkage to representative, contemporary datasets in the 2011 census. Our methodological concern here is to consider how far the sample skew within the GBCS affects its potential to offer a representative mapping of the elite. Is it likely that because the GBCS elicited interest from the well educated and affluent, that the GBCS Elite is unrepresentative of the elite in the wider British population? Here our analysis overlaps with that of the other papers presented in this collection, several of which also examine the micro facets of the GBCS sample skew. In so doing, we will powerfully underscore the utility of GBCS’ identification of an ‘elite’ as a representative or ‘real’ social group.

The second section will move to explain how the Elite class was constructed and can be understood in terms of the constituent cultural, economic and social capital variables available through the GBCS. This will be useful in allowing us to unpack the specific geographical dimensions of these three key variables that we used in constructing our latent class analysis, and this throws up some important findings, especially with respect to the specificity of London.

Finally, the paper will dissect the group further to address the spatial, social, cultural and economic complexity which belies the descriptor ‘Elite’. As one commentator has remarked, the term is inherently ‘recursive’ (Dorling, 2013), with varying Elites in different places, characterized by varying patterns of access, residence, mobility and consumption. The GBCS has remarkable potential to address these concerns, and the final section will shed new light on these patterns in the context of Greater London as a vortex for Elite construction and containment in the UK. The sheer sample size means that this paper can provide a national ecological approach to issues of inequality which Sampson (2012) has argued provides a vital means of understanding social disparities and a methodological counter-balance to the ‘individualistic fallacies’ of traditional sociological scholarship. While this discussion has clearly acknowledged the global character of wealth in London and whilst also recognizing that dynamism and mobility are powerful forces in *uber*-elite formation (Elliott, 2013; Urry, 2013), our focus here for practical reasons is on the capital as a locale for our more expansive conceptualization – the GBCS Elite. In this analysis the focus is on London as a site of elite agglomeration and consumption rather than on its place as a node within a much more nebulous and unquantifiable network of international elite transactions and interactions.

## The end of a ‘modest’ elite? Making sense of the sample skew

The initial GBCS research paper published in April 2013 clearly stated the heavy selection bias in the survey towards university educated and more affluent social groups (Savage *et al*., 2013), with the National Statistics Socio-economic Classification (NS-SEC) I/II groups of senior managers, modern and traditional professionals all being severely over-represented in the BBC web sample. As we recognized, this poses a challenge to using the GBCS for developing a model of the class structure which we addressed by using a small nationally representative survey (GfK) alongside the GBCS. As critics have pointed out, this means we are not fully able to deploy the rich and granular detail provided in the web survey, and it is this issue which we will initially address in this analysis.

Let us begin by noting that the Elite class which we distinguished in Savage *et al*. (2013) is derived using latent class analysis on measures of economic, social and cultural capital. The elite is characterized by its extremely high levels of economic capital, alongside high (though not outstanding) scores for ‘highbrow’ cultural capital as well as social capital. Taking David Rose's (2013) point that it is also possible to derive a more conventional measure of an elite using NS-SEC occupational measures, a starting point in our reflections is to consider the overlap between these two geographies.^1^

**Figure 1:**
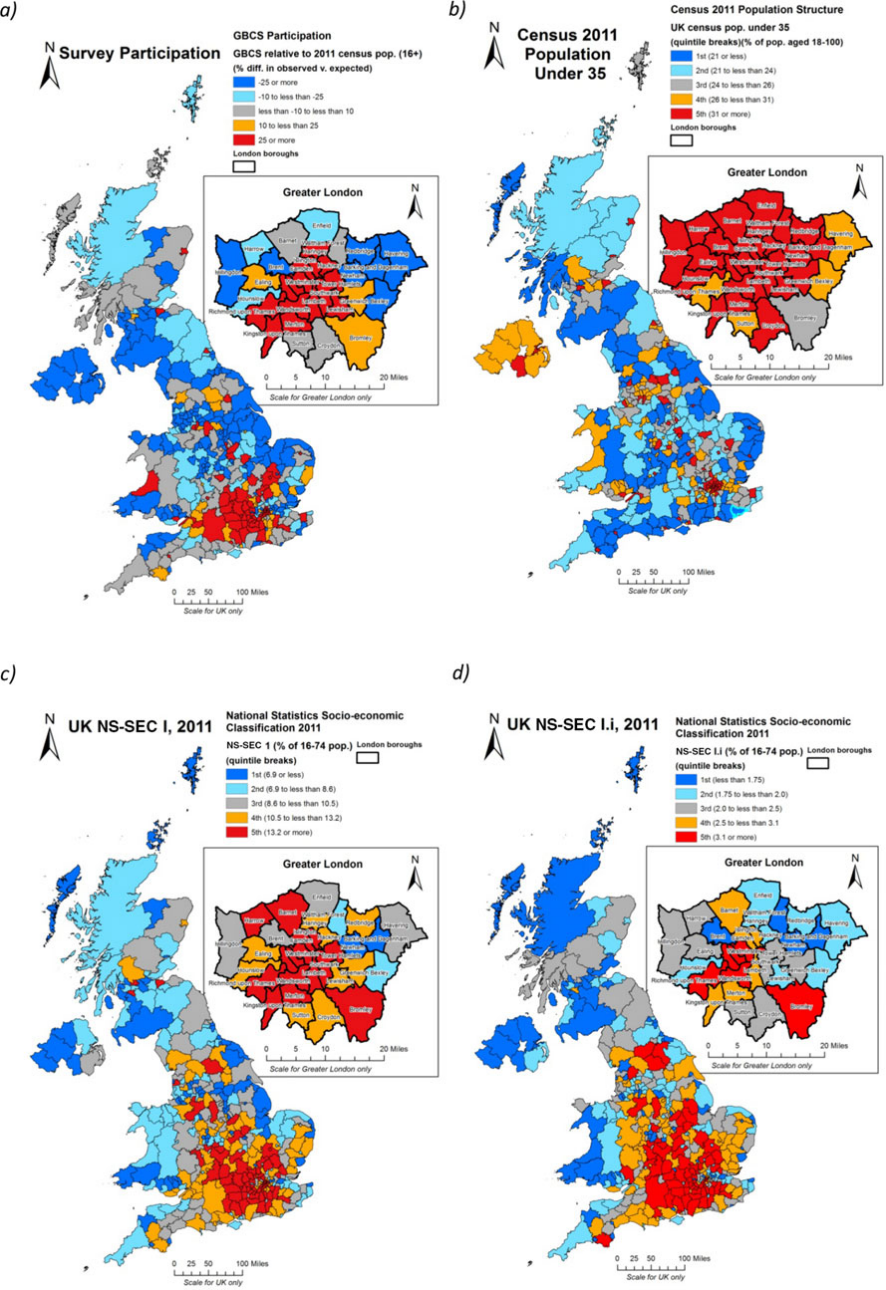
a) GBCS participation relative to the 2011 census population aged 18 and over; b) the census population aged under 35; c) NS-SEC I and; d) NS-SEC I.i populations

Figure1 provides a starting point for our analyses by demonstrating the striking overlaps between the spatial distribution of the GBCS’ Elite and the NS-SEC I population drawn from the 2011 census. NS-SEC I, comprising 12 per cent of the British population is composed of higher managers and professionals. We also include a more restrictive definition of the elite, NS-SEC I.i (Large employers and higher managerial occupations) with about 3 per cent of the workforce. The census data provide an extremely useful comparator not simply because they are based on the complete population and therefore completely representative, but also because the 2011 enumeration was conducted in extremely close temporal proximity to the first wave of data from the GBCS survey, gathered between late January and the end of June 2011. The 2011 census was conducted approximately halfway through this collation period on the night of 27 March.

Responses to the GBCS were skewed not only in socio-economic terms but also in terms of geography. Figure1(a) shows the spatial distribution of respondents to the GBCS by unitary authority in Great Britain and Northern Irish county.^2^ Along with swathes of western Scotland and the north of England, much of Northern Ireland features in the lowest quintile of response areas to the survey (indicated in white/blue) and the response rate was 50 per cent lower than might be expected given the population base. At the other end of the scale, areas over-compensating in terms of response to the survey (indicated in black/red), were clearly heavily clustered in Greater London and the south-east of England more generally, and especially in the areas west of London in the heart of the home counties. We can also see how university towns and cities are likely to figure as being over-represented, with York, Abedeen, Exeter, Canterbury, Aberystwyth and Norwich all standing out from their hinterlands.

In general terms, this provides a remarkably close reflection of the distribution of the UK's NS-SEC I population (Figure1(c)), which as a statutory definition of the ‘elite’ is a profoundly southern formation. Nevertheless, at intra-regional level pronounced disparities are also apparent. In the north of England, this is evident in the ‘islands’ of affluence in Cheshire and in that part of North Yorkshire approximating to the so-called ‘Golden Triangle’ with vertices linking York, Harrogate and plush north-east Leeds (House of Commons, 2006: Ev.317). Here, the distribution of NS-SEC I is strong, though GBCS responses are somewhat less evident. While within Greater London, historic inequalities between the east and west of the capital clearly still persist in contemporary metrics (Dorling *et al*., 2000; Gregory, 2009). A subdivision of NS-SEC I is also mapped (Figure1(d)), identifying ‘large employers and higher managerial and adminstrative occupations’, in essence, an even more exclusive statutory determination of an elite. The south-east bias is again clearly evident although within Greater London the use of NS-SEC I.i appears to discriminate considerably more across the capital.

Broadly, the GBCS itself is considerably over-represented by those living in elite areas, as one would expect given the general skew towards the elite classes. This is further testimony to the performative character of the survey itself, which is a research instrument which interests and engages those who are relatively economically and socially privileged (see further, Savage, 2015). However, we can see interesting exceptions in this broad skew which makes it clear that the GBCS responses are not a simple reflection of elite geographies. There is the notable pattern in settlements outside the south-east in areas with large student and academic populations, groups who responded in disproportionately high numbers to the survey. The concentration of higher-level educational establishments and employment opportunities in urban areas more generally lies behind the broader demographic imbalances that exist between predominatly urban and rural districts, as evidenced in Figure1(b), which shows that the proportion of the census population aged under 35 is also heavily skewed to the UK's towns and cities.

**Figure 2:**
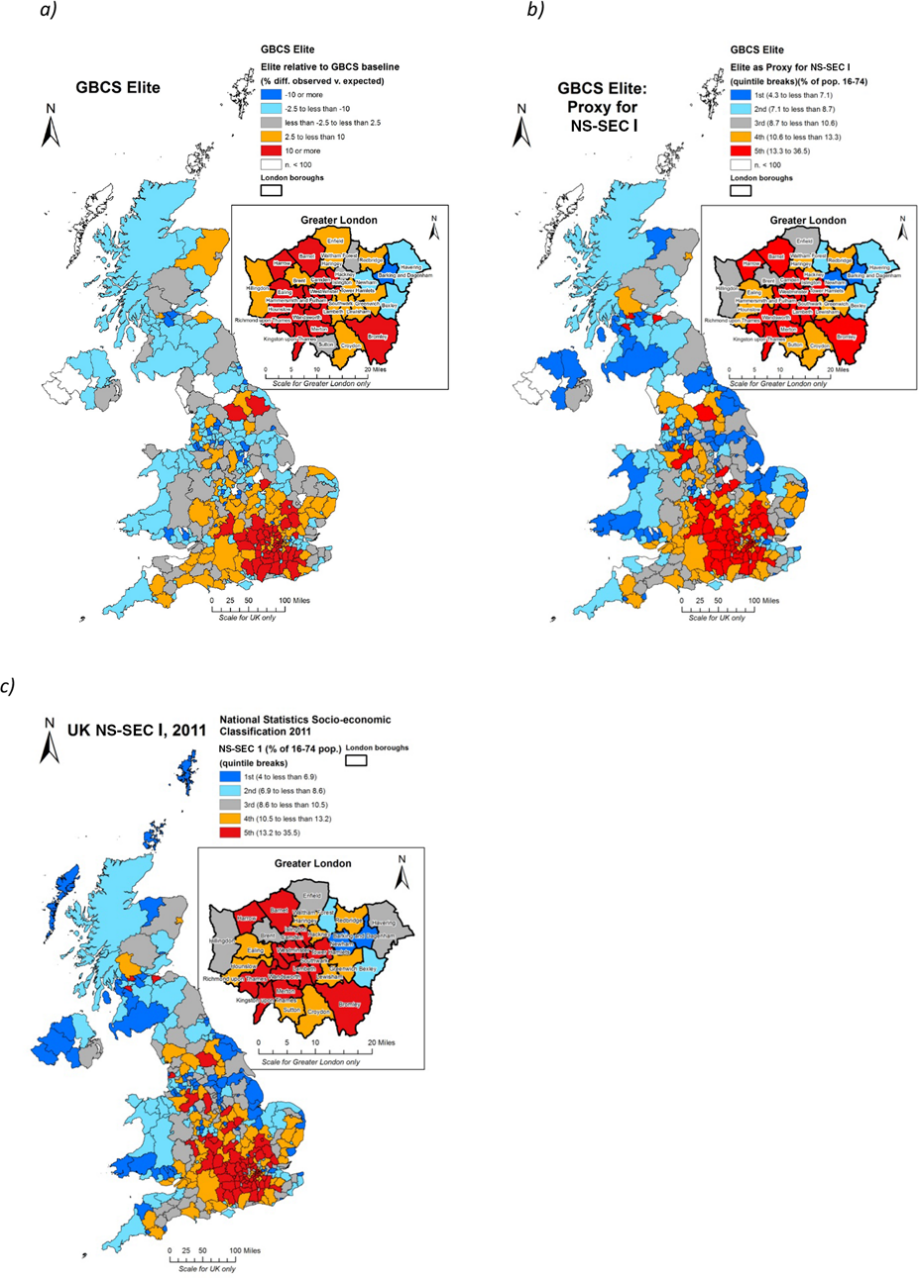
The spatial distribution of the GBCS Elite, a) relative to the GBCS as a whole; b) when used as a proxy for NS-SEC I against the working-age census population; and c) NS-SEC I for reference purposes

**Figure 3:**
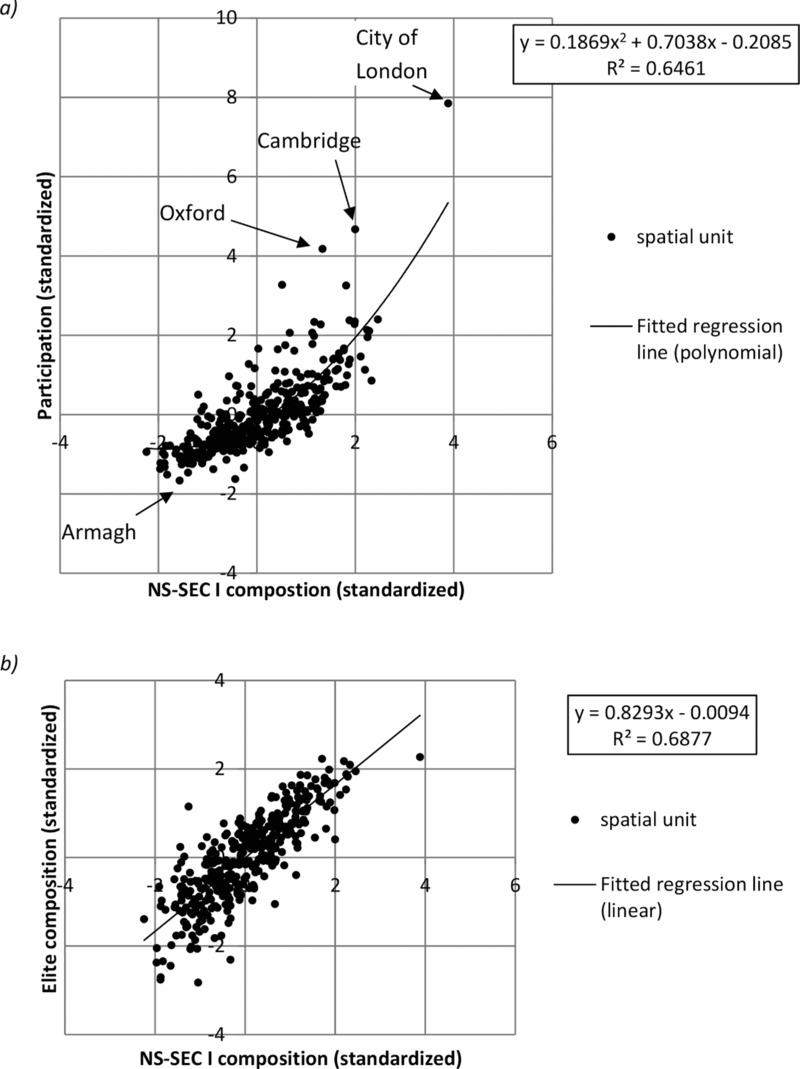
Z score scatterplots of spatial units for a) NS-SEC I population and survey participation rates and; b) NS-SEC I and GBCS Elite populations

Let us now turn to consider the geography of the GBCS elite itself. Figure2(a) shows the distribution of the Elite relative to the GBCS sample as a whole, while Figure2(b) benchmarks Elite respondents from the GBCS against the population as a whole using it as a proxy for NS-SEC I, as derived from the 2011 Census. For ease of comparison, Figure2(c) once again shows the distribution of the census NS-SEC I population from the 2011 census. It is perfectly clear from comparison of Figures2(b and c) that the GBCS Elite presents a geography that is almost uncanny in so closely reflecting that evident solely from the indicators of employment and occupation used to construct the NS-SEC schema. The geography of our Elite class from the GBCS is an almost perfect match with that derived from NS-SEC census data, which in some ways is surprising given that measures of economic capital include house price and savings which are only indirectly linked to occupation. Furthermore, the economic capital measures used in the latent class model from which our Elite was derived were given only equal weighting against the social and cultural input variables. In any event, the overlaps are further borne out in the sequence of graphs in Figure3, which show the relationship between spatial units more precisely. Figure3(a) plots the standardized scores for participation rate in the GBCS against the NS-SEC I composition of each area by spatial unit and show a very close fit. Some of the outliers are highlighted on the graph and are highly revealing. The City of London is globally recognized as a centre of financial transaction and accumulation, a fact reflected in the socio-economic profile of the vast majority of its select group of residents. However, notwithstanding this fact the City still responded to the survey in terms wildly over-proportionate to its wealth. Other areas which exceeded even the quadratic relationship between participation and NS-SEC I composition were, for example, Oxford and Cambridge, highlighting the pronounced bias towards elite university towns in the response pattern (see more generally, Wakeling and Savage, this volume). At the other end of the spectrum, the example of Armagh is an area where both participation rates and NS-SEC I composition were much lower. It is likely that in Northern Ireland, where response rates more generally were much lower than would be predicted by the population base, issues of national identity provided an important interaction effect in determining responses to the ‘Great *British* Class Survey’, with participation rates lower in areas with substantial Catholic populations. It is likely that, in effect, the particular character of the Northern Irish polity, historically structured more around ethnic rather than class distinctions, had a profound role to play in explaining this regional effect (Cunningham, 2013; Gregory *et al*., 2013; McGarry and O'Leary, 1995a; 1995b). In overall terms, a strong relationship existed between the variables, with an *r*^2^ of 0.65 between participation and NS-SEC I composition.

Figure3(b) shows that in terms of the intersection between NS-SEC I and the GBCS Elite, the relationship in distributions was even stronger, again indicating that rather than being an abstract concept, the GBCS Elite closely resembles in spatial terms, formulations of privilege that represent the current academic and statutory benchmark. It is little wonder, then, that standardized values for levels of NS-SEC I, participation and Elite correlate powerfully with *r*^2^ = 0.83 for NS-SEC I and Elite geographies and an *r*^2^ *=* 0.77 for participation and NS-SEC I.^3^ It would seem therefore undeniable that the socio-economic complexion of areas has had a profound bearing on participation levels in the survey, and statistical testing reveals that this relationship was quadratic rather than linear, with the degree of participation increasing with the NS-SEC I proportion of the spatial unit. Using NS-SEC I population as the predictor and participation as the dependent variable in a quadratic curve estimation regression showed that NS-SEC I had a significant effect in predicting participation rates with *β* = 0.76, *t*(361) = 22.59, *p* < 0.01. It also explained a significant degree of variance in the participation rates, *r*^2^ *=* 0.65, *F =* (1, 361)328, *p =* < 0.01.^4^

**Figure 4:**
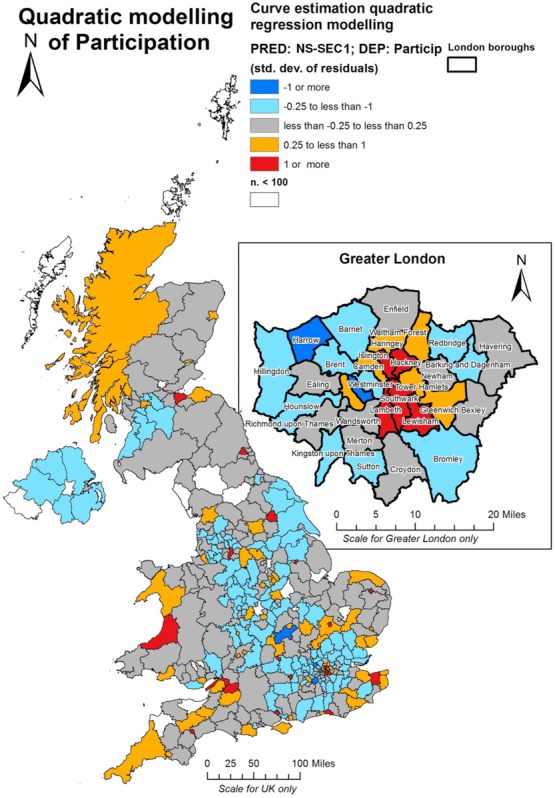
Mapping of residuals from a quadratic curve estimation model with GBCS survey as the dependent variable and NS-SEC I composition as the predictor

Figure4 maps the resulting residuals from the above regression. The purpose of this is to highlight if patterns exist in terms of areas which over-contributed or under-contributed to the GBCS in relation to what we would expect from their NS-SEC I populations. This provides some interesting insights in placing the survey skew into a wider national socio-economic perspective, as fairly large areas of the south-east were moderately under-represented given their socio-economic profiles as predicted in the regression model. In London, the results show that patterns of over-participation in the GBCS were actually rather more concentrated in the boroughs immediately to the south and north of the river Thames in central London, rather than encompassing the western boroughs such as Westminster and Kensington and Chelsea which include some of the most affluent areas in the entire country. There appear to be distinctive ‘London’ effects here which probably reflect the fickle and nuanced imprint of gentrification upon the capital and suggest that it is those with ‘emerging’ cultural capital who might be predisposed towards the GBCS (see Savage *et al*., 2013; Prieur and Savage, 2013; Savage and Hanquinet, 2015). Thus, one of the London boroughs which saw the highest levels of participation is Hackney, an area of extreme and well-established gentrification which has been the focus of much attention for urban geographers (Butler, 1992, 1995, 1997; Butler and Robson, 2003; May, 1996). This is a theme we will return to later on in the paper.

Beyond London, the map also underlines once again the pronounced university town effect earlier noted: Aberystwyth, Brighton, York, Oxford, Cambridge, Manchester, Bristol, Newcastle, Edinburgh, Southampton all come through strongly in this analysis as having GBCS responses higher than one would expect on the basis of their NS-SEC I distributions. It is worth noting that the areas of London with over-representations are also sites of the city's major universities.

## Constructing the Elite

The strong spatial and statistical overlaps between the UK's census social class geographies and the GBCS Elite can be taken as evidence towards the sociological value of the GBCS latent class analysis. This section sheds greater light on the geographies of the constituent economic, social and cultural capitals which were used to construct this concept of the Elite, so we can begin to dissect their geography in greater detail so that we can see if the Elite in different areas of the UK have different social and cultural characteristics. At this point in the analysis, we depart from census data which has no direct information on these issues, and can begin to use the unusual insights from the GBCS with their extensive questions on cultural and social issues.

**Figure 5:**
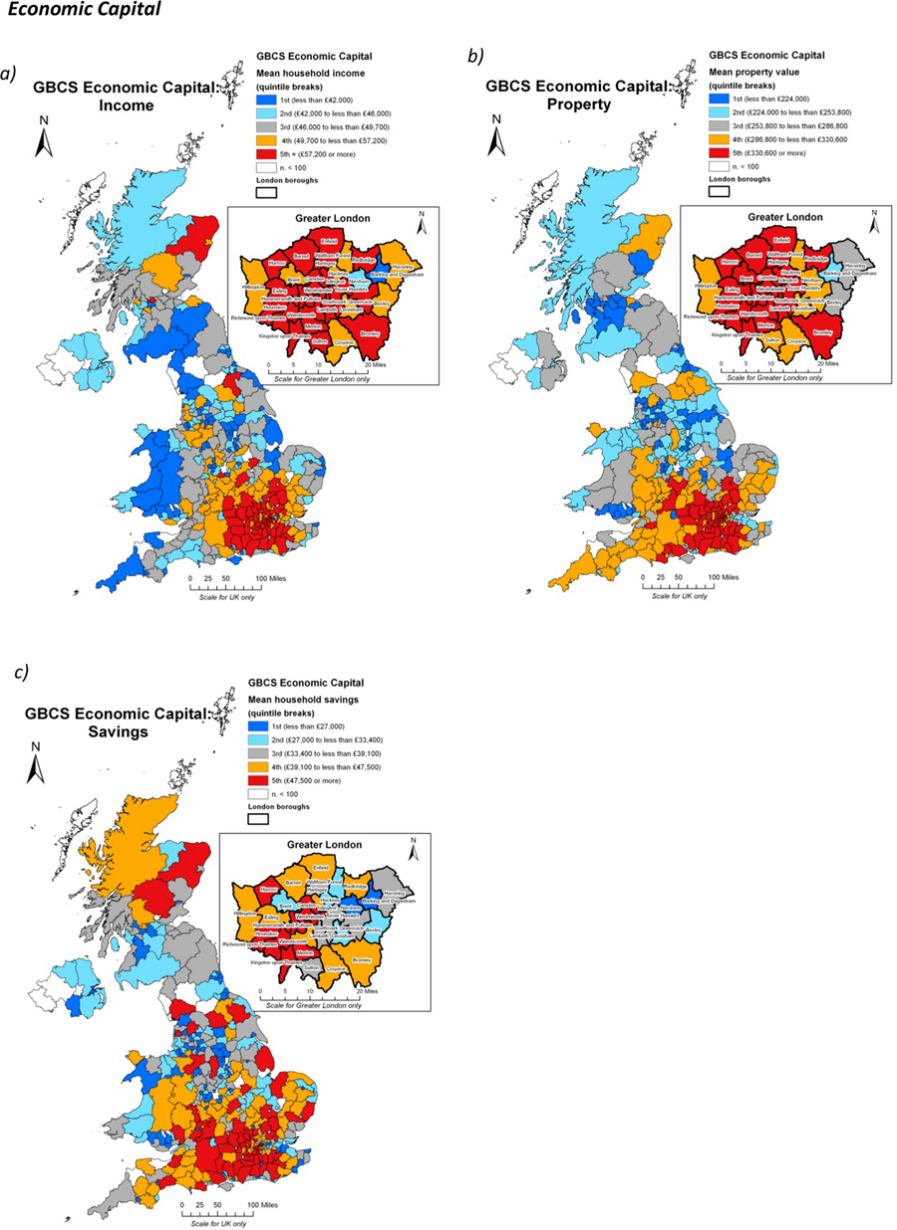
The spatial distributions of the three constituent measures of economic capital in the GBCS; a) mean household income; b) mean property value; and c) mean household savings

First, let us show the map of economic capital. Figure5 presents the geographical distributions of the three constituent measures of economic capital: mean household income, mean property value and mean household savings. Household income and property value (Figure5(a and b)) display similar distributions and are both heavily skewed to London and the south-east, reflecting both the greater opportunities for higher-paid employment in the region as well as the heavy and related underlying bias in the UK property market. We might see the close association between these two maps as a telling indication of the overlap between inequalities in both labour and housing markets. A slightly more dispersed pattern can be observed with regard to household savings (Figure5(c)) though the same general bias towards the south-east is again clearly evident. The patterns of economic resource concentration present an orthodox picture of the UK, but the image becomes more interesting as we turn to the other capitals.

**Figure 6:**
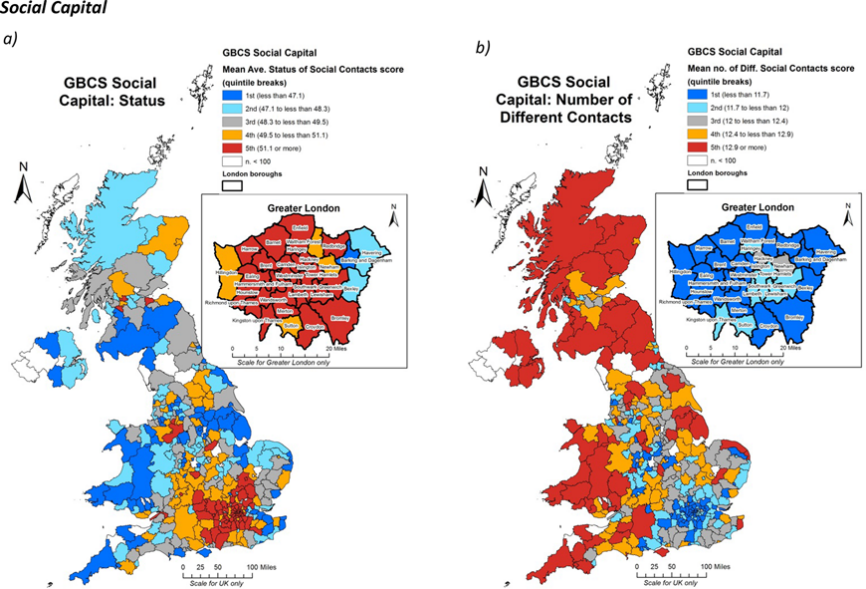
The spatial distributions of the two constituent social capital measures in the GBCS; a) mean averaged status of social contacts; and b) mean size of social networks

The two constituent measures of social capital presented in Figure6 effectively represent mirror images of one another. Figure6(a) shows the spatial distribution of the mean status of a respondent's social contacts averaged across their spatial unit. Areas in black/red therefore indicate those parts of the country where the mean averaged status of the respondents’ social networks were highest and white/light grey/blue where they were lowest. These status scores were calculated using a ‘position generator’ technique in which respondents were asked to state whether or not they knew socially any people from a list of 37 occupational backgrounds (34 of which were used in the analysis). Here, each occupational background was given a score based on the Cambridge Social Interaction and Stratification (CAMSIS) scale. This indicates that there was a greater concentration of people with higher-status social networks in London and the south-east than elsewhere in the country. Furthermore, this pattern appeared to be remarkably clustered once again, closely reflecting the trends for the economic indicators. This distribution was effectively a diametric inversion of the pattern in Figure6(b). This map displays the mean number of people from different occupational backgrounds that people knew socially, thus providing a measure of the socio-economic size of people's social networks. In contrast to Figure6(a), Figure6(b) shows that the number of contacts seemed to increase with distance from the capital. So while in the south-east there was a concentration of higher-status social networks, these networks tended to be far less extensive than was the case as one moved further from the capital. To some extent, this is what one would expect, as if one knows more occupations, their status scores are likely to be deflated as one is largely constrained to know respondents of different occupations with contrasting status scores. Nonetheless, the two measures are not tautologous. It would be possible for those with relatively small social networks to only know those doing routine occupations. Furthermore, given that we have demonstrated how closely rates of participation track representative census indicators, we can be confident that we are mapping substantive socio-spatial distinctions.

**Figure 7:**
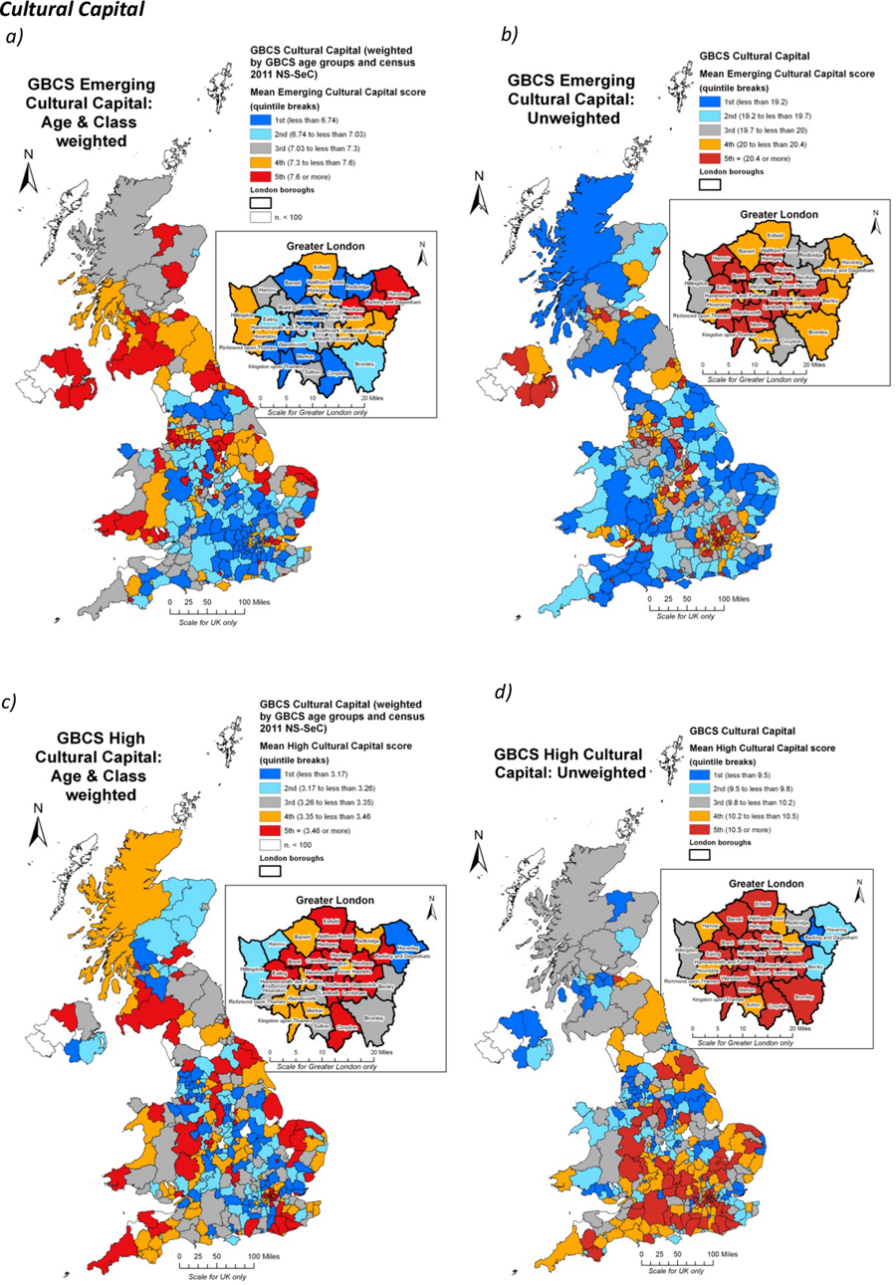
The spatial distributions of the two constituent cultural capital measures in the GBCS: (a and b) emerging; and (c and d) highbrow. The (a and c) maps have been weighted against underlying demographic and socio-economic populations using representative census indicators

Finally, Figure7 presents the two measures of cultural capital, ‘emerging’ (Figure7(a)), and ‘highbrow’ (Figure7(b)). Emerging cultural capital activities include a wide range of everyday pursuits such as playing and watching sport, surfing the Internet, visiting social networking sites and socializing with friends. Highbrow activities were more conventional and traditional forms of cultural engagement, including visits to theatres, museums and stately homes, listening to jazz or classical music or eating at French restaurants. Both of these contrasting indicators emerged as discrete forms of cultural capital as they were differentiated on the second axis of a Multiple Correspondence Analysis (MCA) of the wide range of cultural activity indicators available in the GBCS dataset. One criticism of these indicators lay in the fact that both could be seen to be influenced by underlying demographic factors. In the case of emerging cultural capital, many of the activities identified through the MCA procedure could be seen as tending to be more associated with younger age groups, while the highbrow input variables might well be seen as leaning towards older and more affluent social groups as effectively ‘posh’ pursuits. In essence, there is an inherent danger that the identification of such seemingly discrete modes of engagement is merely modelling underlying demographic and class socio-economic biases towards particular types of cultural engagement. Read carefully, the MCA analysis refutes such criticisms as neither forms of highbrow or emerging cultural capital are the simple product of age, since it is only those who have attended universities and are in managerial and professional groups who are predisposed towards them, and there is also considerable other research pointing to the existence of these different forms of cultural capital (see Prieur and Savage, 2013 for a review and discussion). Furthermore, the association between age and the different modes of cultural capital is integral to their sociological conceptualization. Having made these points, we can now seek to explore the geography of this intersection between the demographics and these forms of cultural capital so that we can better understand their constitution.

Figure1(b) has already shown that urban areas tend to have higher populations of young people than predominantly rural districts and it likely therefore that any mapping of emerging cultural capital will bias towards towns and cities. To counter that problem, the emerging (Figure7(a)) and highbrow (Figure7(c)) indicators mapped here have been weighted against the underlying age structures and class distributions available from the representative census sample.^5^ When both class and age have been controlled for, emerging cultural capital shows a distinctive concentration in the ex-industrial heartlands of northern England, Northern Ireland and parts of Scotland, with lower scores in much of the south-east with the exception of parts of East London and the Thames Gateway. West London and the Home Counties no longer appear to be a centre for such cultural forms, and instead it is the cultural vibrancy of the northern English cities (in the red belt stretching from Merseyside into Yorkshire), and some of the Scottish, Welsh and Irish districts which appear striking.

By contrast, highbrow cultural capital is much more dispersed when the data are weighted, but a bias towards the inner boroughs of London can still be discerned. Across the country as a whole, a weighted approach suggests a much less conventional relationship with the geographies of economic affluence than might otherwise have been predicted and points to a distinctive urban and geographical quality to forms of cultural capital that cannot be reduced simply to the demographic or class characteristics of these places.

## London as ‘elite metropolitan vortex’

By this stage it should have become apparent that spatial complexity is integral not simply to an understanding of the relationship between different groups and classes, but also within them. Using spatial analysis techniques we can shed further light on the matryoshkan quality of the Elite and the power of geography in the group's formation because traditional statistical approaches to class analysis have tended to rely on global measures, assuming that underlying relationships tend not to vary across space (Gregory and Ell, 2007: 163). In the remaining part of the paper, we are especially interested in unravelling the relationship between the metropolis of London and aspects of Elite formation. We are interested here in pursuing the idea that the contemporary elite is necessarily a metropolitan class, that is, that its geography is not an incidental by-product of where it happens to reside, but, by contrast, that the London metropolitan area is itself a vortex in which a distinctive elite formation is generated. The spatial proximity of relatively large numbers of Elite individuals is implicated in the development of distinctive cultural and social, as well as economic patterns which appear distinctive to the metropolitan Elite. To put this in terms of complexity theory, we might be able to detect forms of elite ‘emergence’ in the city which cannot be found elsewhere. Insofar as this is the case, it is powerful repudiation of the claims of writers such as Castells (1996), Bauman (2007) that elites are somehow a ‘place-less’, global formation. As the early findings from Burrows’ (2013a, 2013b) work also attests, there is clear evidence of a strong sense of territoriality within our Elite and spatial propinquity may therefore not be as irrelevant to this group as Webber's (1964) earlier work also suggested. Webber saw such parochial concerns as the preserve of the working class, who had such an investment of economic, social, cultural and familial resources within their immediate communities.^6^ This corresponds neatly to Savage's (2010) assertion that *contra* Castells and Webber, space is crucial not only to an understanding of middle-class identity in the form of ‘elective belonging’, but must be integral to any broader analysis of the factors which act to construct social class more generally (Savage, 1996). From a Bourdeusian (2000) perspective, these processes of gentrification are a clear manifestation of the construction of *habitus*, an urge for socio-spatial security and stability. In terms of the disorientating impacts of globalization, the enduring, indeed increasing power of locality is testament to a very human desire for a kind of socio-spatial anchorage to mitigate against its centripetal cultural effects (Savage *et al.*, 2005).

**Figure 8:**
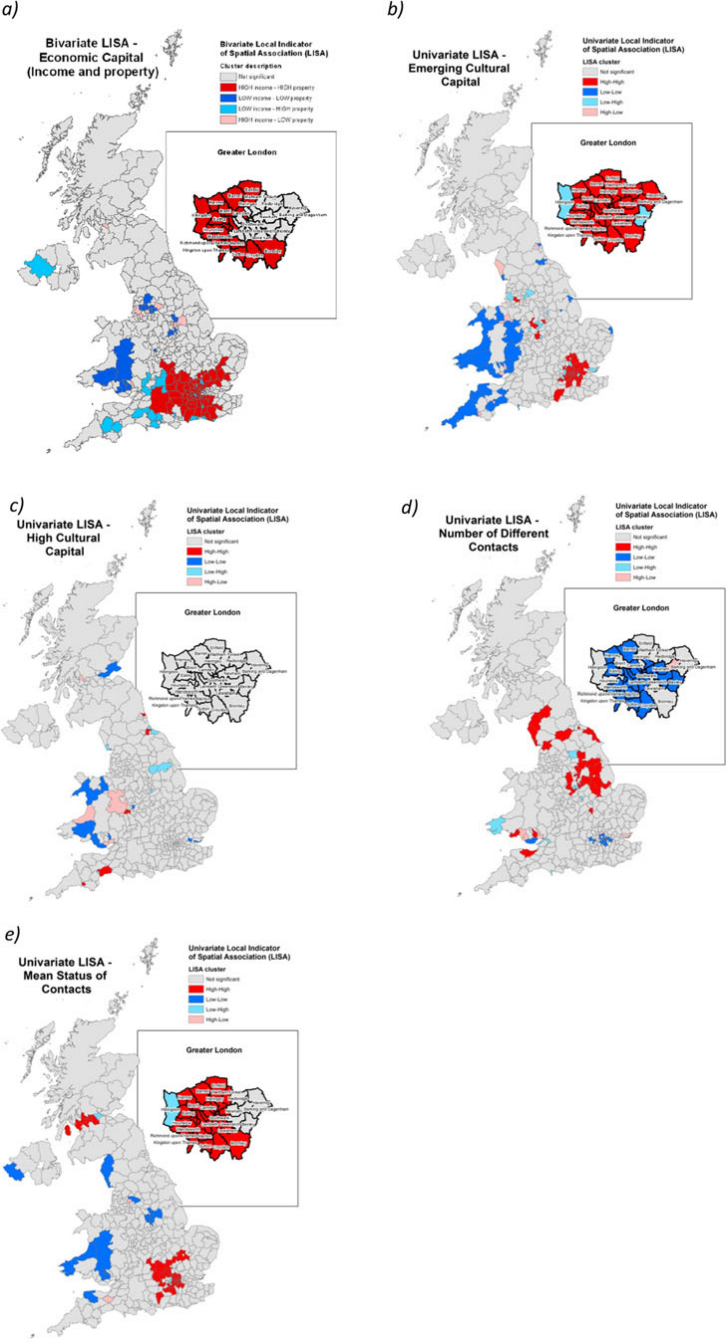
Local Indicator of Spatial Association (LISA) clustering of the GBCS Elite by: a) economic capital; b) emerging cultural capital; c) highbrow cultural capital; d) number of different contacts in social network, and e) mean status of social network

Figure8(b–e) presents the results of a univariate Local Indicator of Spatial Association (LISA) analysis of the various input variables used in the construction of the latent class model in the original GBCS analysis. The technique works by identifying statistically significant clusters using a local regression methodology (Anselin, 1995; O'Sullivan and Unwin, 2003). LISA has an advantage over other spatial clustering techniques such as Moran's *I* in being able to identify not only where high and low concentrations exist, but also where outliers of either extreme values are surrounded by opposing scores. The analysis thus returns four possible outcomes: areas of high values surrounded by other high values (‘High-High’ clusters), areas of low values surrounded by other low values (‘Low-Low’ clusters), outliers of high value surrounded by low values (‘High-Low’) and the inverse pattern (‘Low-High’).

Figures8(b and c) show the results of LISA analysis on the cultural variables based solely on the Elite. Within the Elite, London and parts of the south-east stand out as important in the formation of emerging cultural capital, while rural and more distant parts of Wales and the south-west indicate clustering of lower values within the class. In contrast, highbrow cultural capital (Figure8(c)) does not appear to discriminate to anything like the same extent within the Elite as no discernible clusters emerge. This evidence suggests there are distinctive patterns of cultural engagement within the London Elite which are not found elsewhere, testifying to the city as cultural vortex for this group.

Social capital (Figures8(d and e), on the other hand, does once again appear to show a much more pronounced pattern and one that reflects the wider trend within the GBCS (Figure6) towards more enclosed social networks of higher status in the south-east versus wider and a lower mean status of social contacts as one moves further from London.

In Figure8(a) an adaptation on the technique has been applied showing the results of a bivariate LISA analysis which acts to cluster the data along two axes (in this case, mean household income and mean property value). Perhaps more than any other map, it is this which highlights the profound stratification that exists within the Elite. Economic power within this group is exclusively clustered in the south-east of England (black/red), where property values in addition to higher average income levels act to mark out a sub-group of elevated financial advantage. In contrast, grey areas identify what we might loosely define as the ‘poor Elite’ in Wales and parts of northern England. The outliers are also interesting in throwing a fascinating light on the relative and spatially contingent nature of the Elite with clusters around Lancashire and South Yorkshire, where incomes are high but property assets relatively modest (backslash hatch/light blue). While in and around the south-east we can also see the inverse pattern in the slashed hatch areas as property values fall away on the cusp of the metropolitan economic vortex.

A further way of exploring the structure of the elite within London, building upon this recognition of its cultural, economic and social specificity is to break it down into three distinctive types, namely the business, cultural and legal groups. There are 1,431 members of the cultural Elite, determined as those in the Elite latent class who also have the following and related occupations: actors, artists, arts officers, dancers, higher education teaching professionals, journalists, editors, photographers, authors, conservation professionals and musicians. This compares with no less than 3,000 chief executives (members of our Elite latent class, living in London). Finally, we can distinguish a distinctive legal Elite constituted by barristers, judges, legal professionals and senior police officers (*n* = 404).

**Figure 9:**
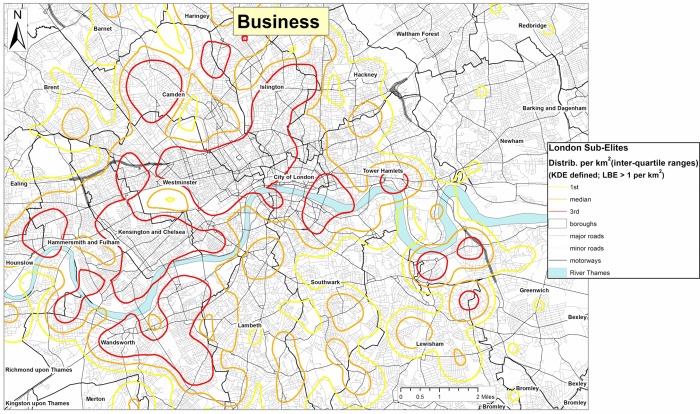
Residential geographies of the London Business Elite

**Figure 10:**
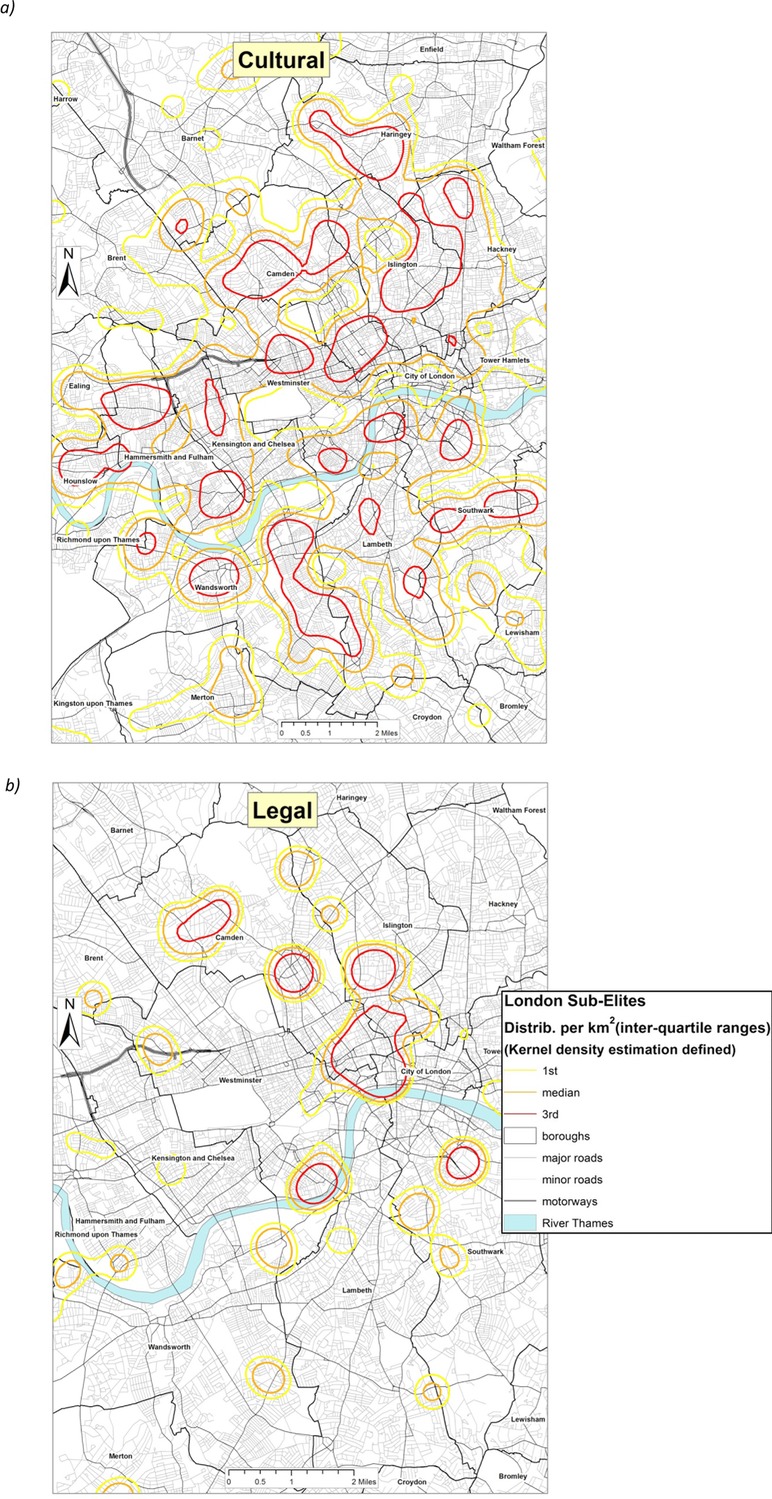
Residential geographies of the a) London Cultural and b) London Legal Elites.

Figures9 and 10 examine the geography of these different kinds of Elite within London, showing a partly overlapping, but partly differentiated geography between them. The business Elite (Figure9) – the largest and most affluent sector of the London Elite – are most likely to be located in the western heartlands of London, centred on Westminster, Kensington and Chelsea, and stretching north through Camden and Islington, and with outliers in Greenwich and Richmond. The legal Elite (Figure10(b)) by contrast, is centred more on the Eastern centre of London (closer to the law courts) notably in the City of London itself, and reaching south of the river into Southwark. The cultural Elite (Figure10(c)) is the most spatially dispersed, and least likely to be located in the western heartlands of the London Elite. There is a substantial north London contingent centred in Haringey, Islington and Camden; a southern London grouping located in the Borough of Wandsworth (Battersea, Balham and Putney), and more suburban clusters in Ealing and Hammersmith (close to the BBC studios at White City where some of their number might be employed). We can thus detect a distinctive Elite micro-geography within London, partly explicable in terms of the economic assets of the different groups (with the business Elite being the most affluent), partly by the geography of employment, but also possibly through the spatio-cultural preferences of these groups themselves. Affluent incomers have traditionally been drawn to the inner-city by an urban aesthetic of ethnic diversity and working-class authenticity which their presence ironically and systematically erodes. Thus, in no small way do these maps reflect the wider social, cultural and economic dynamism of the capital's micro-geographies and the transformative power of gentrification in particular parts of London (Pratt, 2009).

**Figure 11:**
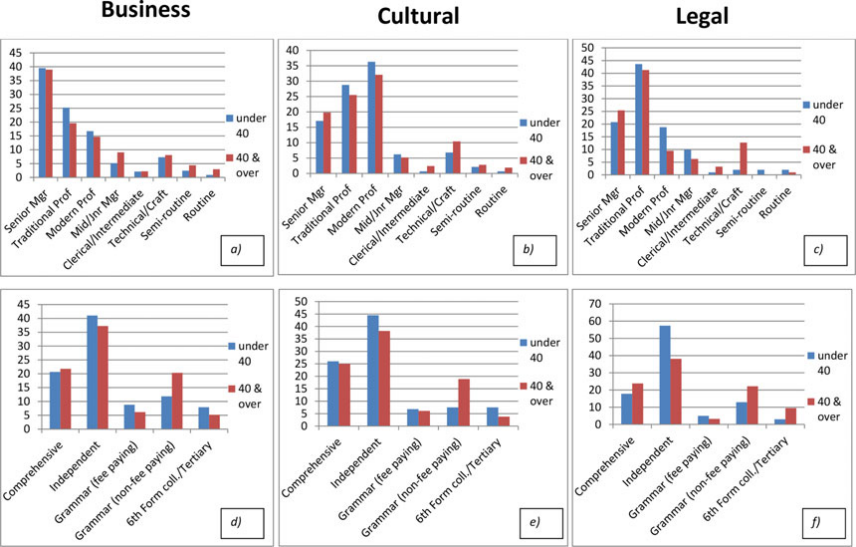
Changing patterns of recruitment into the London Business, Cultural and Legal Elites by parental class (a, b and c), and by secondary educational establishment type (d, e and f)

Figure11 further explicates the intriguingly different demographic composition of these three London Elites, comparing the Elites aged over and under 40. This allows some comparison of change over time, though it is important to recognize that age, cohort and period effects are confused here. All show a propensity towards self-recruitment, with a relatively large proportion of the business Elite having a senior managerial family background (40 per cent, compared to around 20 per cent for the other two Elite groups), a relatively large proportion of the cultural elite coming from ‘modern professional’ backgrounds (around 30 per cent), around double the proportion from the other two groups, and the legal Elites with a high proportion from traditional professions (many of whom might be lawyers). Interestingly, these trends towards self-recruitment are not declining.^7^ Recruitment from routine and semi-routine occupations is very small indeed, which indicates the extent of the exclusiveness of this London Elite.

The patterns with respect to schooling are also striking. Around 40 per cent of all these elite groups are recruited from independent schools, and this proportion is somewhat higher for the younger age groups. This is largely explicable in terms of the declining proportion of the younger age group recruited from grammar schools, but Figure11 shows this slack is taken up by 6th form colleges and independent schools rather than comprehensive schools, whose share of recruitment into all three Elite groups is falling slightly.

## Conclusions

We should begin our conclusion by reiterating that since the GBCS is not a representative sample, we are not in a position to establish definitive and causal relationships about the relation between geography and social class. Nonetheless, we have shown patterns which we are satisfied are highly revealing. In fact, the very geography of the sample skew is itself a fascinating indicator of how the GBCS plays into an elite sensibility. We also hope to have shown that because of its large sample size and granularity, the spatial analysis of the GBCS can suggest striking features of contemporary elite formation which are not readily discernible from other data sources.

Our main argument has been to suggest that GBCS data indicates how London can be seen as an ‘Elite metropolitan vortex’. Within the UK, the elite, whether as defined by our latent classes, or by NS-SEC I, are overwhelmingly located in London and the south-east of England, and this geography plays a central role in their formation: it is not incidental to who they are. The concentration of such Elites within London explains why their social capital tends towards higher status contacts, and is hence more exclusive, and tends towards greater sociability with other Elite members. By contrast, we doubt that other urban locations, even including Edinburgh, act as a zone of elite formation even in a more modest way. It is the distinctive combination of employment opportunities, the housing market, and the elite educational system which allows London to be a spatial vortex for the elites to emerge. We are therefore doubtful of those who claim that elites operate beyond ‘place’. While Bridge (1993) has argued that the impact of gentrification on the urban social landscape is not a simple one and is cross-cut by gender, ethnicity and life-stage, we nonetheless believe that it is the patterns of closure which are most striking. We can deduce patterns from the spatial evidence which imply that the Elite have a distinctive geographical footprint which is consistent with their having a clear spatial and social identity.

We have also shown that there is no simple unitary London Elite. Different sections of the Elite can be distinguished, each having a specific pattern of recruitment and micro-geography within the city. We are hence not talking about a cohesive ‘old boy's club’, and the boundaries of this group go rather wider than conventional and common-sensical understandings of elites. The distinctive micro-geographies, however, also suggest that London is a site in which these different groups can both find a habitus where they meet others like themselves, and also co-exist close to other elite sectors. Thinking in these terms allows us to see London as a highly specific spatial vortex where a range of powerful and economically privileged elite agents can potentially access each other whilst preserving also a distinct sub-culture of their own.

The images which appear in this article are referred to as being in colour – this is how they appear online. However, in the hard copy they will appear in black and white.

## Notes

1 See further, on the relationship between NS-SEC measures of class and those used in the GBCS, Savage (this volume).

2 Spatial units with less than 100 GBCS respondents have been suppressed. The reason for the use of a more aggregate level of geography in Northern Ireland was the markedly lower level of response in comparison to other regions of the UK.

3 The correlation between the GBCS Elite and NS-SEC I was stronger than with the more exclusive NS-SEC I.i, which returned an *r*^2^ of 0.76, *p* = < 0.001.

4 Removing City of London from the equation returned an *r*^2^ of 0.60.

5 Two weights were derived from the census; the first, to control for younger and more affluent populations based on the shares of the local population aged under 35 and in NS-SEC I, was applied to emerging cultural capital. The second controlled for older age groups and more affluent populations by identifying the share of the population aged 50 or over and the proportion in NS-SEC I and was applied to highbrow cultural capital. The aggregate standardized or *z* score for each of these two sets of inputs was then used to weight against the respective raw cultural capital scores. The application of such denominators to the raw data has the effect of narrowing the range of score. To assess whether the range of values was still significantly different from 0, the resulting weighted emerging and highbrow cultural capital scores were measured in a *t*-test with a significant result for emerging (*t*(361) = 264.8, *p* < 0.01) and also for highbrow (*t*(361) = 319.3, *p* < 0.01).

6 In a comparative European context, work by Andreotti and Le Galès (2008) has identified a similar sense of deep socio-spatial embeddedness amongst the middle class in Milan and Paris.

7 Though given the marginal shifts in the proportions of the population in these different occupations, it is likely that the relative rates of self-recruitment are falling somewhat. We also need to bear in mind that it might take longer for the children of lower social classes to move into elite positions and hence the differences are to be understood as age effects.

## Notes

Please quote the article DOI when citing SR content, including monographs. Article DOIs and “How to Cite” information can be found alongside the online version of each article within Wiley Online Library. All articles published within the SR (including monograph content) are included within the ISI Journal Citation Reports® Social Science Citation Index.
